# Overexpression of ADAM9 decreases radiosensitivity of hepatocellular carcinoma cell by activating autophagy

**DOI:** 10.1080/21655979.2021.1965694

**Published:** 2021-09-16

**Authors:** Lijin Zhu, Yuanyuan Zhao, Li Yu, Xinjia He, Yingju Wang, Peng Jiang, Rong Yu, Wei Li, Bin Dong, Xiang Wang, Yinying Dong

**Affiliations:** a Department Of Tumor Radiotherapy, The Affiliated Hospital of Qingdao University, Qingdao, Shandong, China; b Center Of Stomatology, Qingdao Municipal Hospital Affiliated To Qingdao University, Qingdao, Shandong, China; c Radiotherapy Technology Center Of The Affiliated Hospital Of Qingdao University, Qingdao, Shandong, China; d Department of Otolaryngology, Jimo District People’s Hospital, Qingdao, Shandong, China

**Keywords:** HCC, ADAM9, radiosensitivity, autophagy

## Abstract

A disintegrin and a metalloprotease (ADAM)9 upregulated within human hepatocellular carcinoma (HCC) cells, but its effect on HCC radiosensitivity remains unknown. The present work aimed to examine the effect of ADAM9 on HCC radiosensitivity and to reveal its possible mechanism, which may be helpful in identifying a potential therapeutic strategy. Changes in ADAM9 expression after X-ray irradiation were identified using western blot, qRT-PCR, and immunofluorescence. ADAM9 stable knockdown and overexpression cell lines were constructed using lentivirus packaging. The radiosensitivity of HCC cells with altered ADAM9 expression was examined by CCK-8 assays, subcutaneous tumorigenesis experiments, and clone formation assays. This study also determined how autophagy affected HCC cell radiosensitivity. Furthermore, ADAM9, p62 and Bax expressions in HCC tissues that were removed after radiotherapy were detected by immunohistochemistry, and the relationship among the levels of these molecules was statistically analyzed. The level of ADAM9expression in HCC cells increased after X-ray irradiation. Through CCK-8 assays, subcutaneous tumorigenesis experiments, and clone formation assays, this work discovered the increased MHCC97H cell radiosensitivity after ADAM9 knockdown, and the radiosensitivity of Huh7 cells decreased after the overexpression of ADAM9. Furthermore, ADAM9 induced HCC cell autophagy via downregulating Nrf2 expression, while autophagy inhibition or induction reversed the effects of altered ADAM9 expression on radiosensitivity. Moreover, ADAM9 level showed a negative correlation with Bax and p62 expression within HCC tissues after radiotherapy. Taken together, ADAM9 decreased the radiosensitivity of HCC cells, and autophagy mediated this process.

## 1. Introduction

Hepatocellular carcinoma (HCC) occupies 90% of the primary liver cancers, which represents a major threat to health worldwide [1]. HCC is a moderately radiosensitive tumor, and the liver is a radiosensitive organ [2–4]. A growing body of literature supports radiotherapy as an effective local treatment for HCC [5–11]. Because of the poor liver tolerance as well as subsequent radiation-induced liver disease (RILD), the therapeutic effect of radiotherapy is poor on treating HCC [12]. Therefore, finding an approach to increase the radiosensitivity of HCC in clinical and fundamental research is of paramount importance.

A disintegrin and metalloprotease (ADAM)9, one of the modular type I transmembrane proteins, possesses one disintegrin-like domain and one metalloprotease [10,11]. ADAM9 over-expression has been suggested to be associated with HCC clinicopathological characteristics, and ADAM9 overexpression potentially results in tumor development, invasion, metastasis and poor prognosis [13–15]. Immunohistochemical and western blot analyses confirmed that ADAM9 is upregulated in human liver cancer cells, and ADAM9 causes reactive oxygen species (ROS) generation through the interaction with NADPH oxidase, promoting the liver cancer cell metastasis and epithelial–mesenchymal transition (EMT) [13,16]. Nonetheless, as far as we know, little research is available on the ADAM9 role in HCC radiosensitivity.

Autophagy is a strictly regulated process by which cell contents are degraded and recycled, and autophagy is an important mechanism of programmed cell death [17]. Autophagy exerts a vital part in cell proliferation, growth, homeostasis and differentiation [18]. The molecular control of autophagy is an elaborate multistep process, mainly regulated by conserved autophagy-related genes (ATG genes), which can form several functional complexes associated with other regulators involved in autophagy initiation and execution. For example, the ATG5-ATG12-ATG6L1 complex acts as an E3-like enzyme to catalyze the conjugation of LC3-I with phosphatidylethanolamine (PE) to produce LC3-II. The conversion from LC3-I to LC3-II is essential in autophagosome formation, and the evaluation of LC3II/I ratio is widely used as marker of autophagic flux [19]. The recruitment of Beclin-1 is effective, after its release from the complex formed with the anti-apoptotic protein Bcl-XL. The free Beclin-1 can induce the sequential recruitment of ATG12-ATG5 promoting conjugation of ATG8 (LC3) to phosphatidylethanolamine in autophagosome, leading to its extension and closure [20]. Various studies have found that autophagy can influence the sensitivity of many cancers to radiotherapy. Wang et al. found that chloroquine can promote bladder cancer cell radiosensitivity and slow radiation-induced DNA damage repair through accelerating apoptosis and suppressing autophagy [21]. Huang and colleagues suggested that MST4 protein level within glioma cells increased after X-ray irradiation, and MST4 enhanced its activity by the phosphorylation of ATG4B, an autophagy-related protein, for inducing autophagy while reducing glioma cell radiosensitivity [22]. In summary, various factors can regulate the occurrence and development of autophagy by influencing key protein molecules in this process and then regulate the radiosensitivity of cells. Therefore, scientifically regulating the level of autophagy may be a means for improving radiotherapy effect.

In this work, we described the first evidence that the induced expression of ADAM9 by X-ray irradiation decreased HCC radiosensitivity, autophagy mediates this process. We also identified a crucial role of Nrf2 in ADAM9 promoting autophagy. Furthermore, we showed a negative correlation between ADAM9 and Bax, p62. Taken together, the overexpression of ADAM9 stimulated by X-ray irradiation could decrease HCC radiosensitivity, and may therefore serve as a therapeutic target.

## 2. Materials and methods

### 2.1. Cell culture and chemicals

Human HCC MHCC97H cells with high malignancy grade were provided by the Chinese Academy of Sciences (Shanghai, China). Huh7 cells with low malignancy grade were purchased from American Type Culture Collection (ATCC) (Manassas, VA, USA). Then, the above two cell lines were cultivated within DMEM (GIBCO BRL, Grand Island, NY) that contained 10% FBS as well as 1% penicillin and streptomycin. The medium was refreshed every 2–3 days and incubated within the humid incubator under 37°C and 5%CO2 conditions, followed by cell passage after reaching about 90%. The cells were trypsinized with 0.25% trypsin (HyClone, Logan, UT, USA) subsequently. Rapamycin and 3-MA were provided by Sigma (StLouis, MO, USA).

### 2.2. Radiation

Cells were exposed to X-ray irradiation at 2–6 Gy within one single fraction by adopting the linear accelerator (Oncor; Siemens, Munich, Germany). After reaching about 75% confluency within the T75 flask, we put the flask on the couch and corrected radiation distribution using the 1.5-cmbolus. The following radiation parameters were adopted: the 3 Gy/min dose-rate, the 6-MV photon beam energy, the 100-cm source-surface distance, the 20 × 20 cm^2^ radiation field size, and the 180°gantry. The cylindrical ionization chamber was used to measure dosimetry before radiation [15].

### 2.3. Patients

The present work recruited 28 HCC cases according to the previous description [15]. From January 2005 to May 2013, 28 HCC cases received hepatectomy at the Liver Cancer Institute of Zhongshan Hospital Affiliated to Fudan University at 2 months following liver radiotherapy. No case underwent chemotherapy prior to hepatectomy.

### 2.4. Lentivirus, and shRNA transfection

Lentivirus construction and cell transfection were performed as previously described [16]. pGCSIL-shRNA-ADAM9 lentiviral vector (shRNA; 5'-AACTGCAGGAATGGCATT-3') and negative control (NC) lentiviral vector that contained the non-silencing shRNA (5'-TTCTCCGAACGTGTCACGT-3') were provided by Shanghai Genechem Company Co. Ltd (Shanghai, China). pGC-FU-ADAM9 cDNA lentiviral vector and pcDNA3. 1 vector that contained the FLAG-labeled ADAM9 were provided by Shanghai Genomeditech Co. Ltd (Shanghai, China). Thereafter, we screened the transfected cells into the G418 (800 mg/mL, Sigma Aldrich, St. Louis, MO, USA) for 3–5 weeks of screening. Clones with stable transfection were verified through qRT-PCR as well as WB assays.

### 2.5. qRT-PCR

As previous work described [16], TRIzol® reagent (Invitrogen, Carlsbad, CA, USA) was utilized to extract the total cellular RNA in accordance with specific instructions. Subsequently, cDNA was prepared from the isolated total RNA (1 μg) through reverse transcription by the use of Superscript First-Strand Synthesis System (Thermo Scientific, Epsom, UK). Afterward, the SYBR Premix Ex Taq (TaKaRa, Dalian, China) was utilized for qRT-PCR using the ABI StepOne Plus Real-time PCR system (Applied Biosystems, Foster City, CA, USA). Relative ADAM9 level was calculated using 2^−ΔΔCt^approach. Primer sequences used were shown below, ADAM9 (5'-CACTGTGAAAATGGCTGGGCTC-3',forward;5'-ACCAGAAGTCCGTCCCTCAATG-3',reverse);GAPDH (5'-CACCATCTTCCAGGAGCGAG-3',forward;5'-TCACGCCACAGTTTCCCGGA-3',reverse).

### 2.6. Western blot assay

Total tissue or cellular protein was extracted using RIPA buffer (Beyotime, Beijing, China). Later, the extracted protein content was detected according to instructions using BCA protein detection kit (Beyotime, Beijing, China). Aliquots of proteins (50 μg/lane) were isolated with 10% SDS-PAGE and moved to PVDF Film (Biyuntian, Beijing, China). And then close the membrane with 5% skim milk for 1 h, followed by incubation using antibodies (Abcam), including anti-ADAM9 (ab186833, 1:1000), anti-GAPDH (ab9485, 1:3000), anti-LC3A/B (ab128025, 1:3000) and anti-SQSTM1/p62 (ab109012, 1:5000), antibodies under 4°C overnight. TBSA-diluted anti-rabbit secondary antibody (Abcam, ab125900, 1:1000) was used for further incubation. The electrochemiluminescence kit (Thermo, Waltham, MA, USA) was utilized to detect the target protein level, which was calculated using the ImageJ software [16].

### 2.7. Immunofluorescence (IF) assay

This study conducted IF staining according to previous report [16]. ADAM9antibody (1:25, Santa Cruz, USA) was dissolved with 1% bovine serum albumin (BSA). The Alexa Fluor 488-labeled goat anti-mouse secondary antibody (Molecular Probes, Eugene, OR) was also adopted for incubation. The images were captured under high-power magnification (х200) by confocal microscopy (Leica Microsystems Imaging Solutions, Cambridge, UK) with identical setting parameters.

### 2.8. CCK-8 assay

After preparing cell suspension, we screened logarithmic growth cells and added them to 96-well culture plates at 2 × 10 ^4^ /mL/well. CCK-8 reagent was added at 10 μl/hole and fresh complete medium was added at 100 μl to incubate for 0, 24, 48, and 72 h, incubate in a 37°C, 5%CO2 incubator. After 4 h incubation, we use an enzyme marker to detect the absorption luminance (OD value) at the wavelength of 450 nm, record the value and draw a growth curve [15].

### 2.9. Experiment of AGAR cloning formation

The logarithmic phase of cell preparation and even single cell suspension, and living cells count, put a layer of 0.6% AGAR in six orifice plate bottom glue, after waiting for the bottom glue fully solidified (about 1 h), respectively, each cell according to the 3 × 10^4^/hole concentration mixed with 0.3% AGAR upper gum, after mixing quickly spread on the lower rubber, after the upper AGAR solidification, under 5% CO_2_, 37°Ccultivation in the box. Later, we added a 0.5 ml DMEM that contained 10% FBS at an interval of 2 days. After 14 days of culture, 1 ml 0.05% crystal violet was added to each well for more than 1 h for staining. Pictures were taken and counted under an inverted microscope [15].

### 2.10. Subcutaneous tumor formation in nude mice

Twenty-four male nude mice of SPF grade (no specific pathogen) 4–6 weeks old were purchased to adapt to the new environment for 1 week. The logarithmic growth cells were washed with serum-free medium for 2 times after X-ray irradiation, and the single cell suspension was prepared. Each nude mouse was given a subcutaneous injection of 0.1 mL cell suspension that contained 1 × 10^7^Huh7 cells or 5 × 10^6^MHCC97H cells in the upper left flank region. Tumor width and length at the site of inoculation were measured to evaluate tumor growth rate. At 10 days later, each tumor was dissected and fixed with 4% formaldehyde to analyze the pathology [16].

### 2.11. Immunohistochemistry (IHC) assay

This study conducted IHC staining according to prior depiction [16]. After paraffin embedding, tissues were sliced into 4-mm sections, washed and blocked using goat serum under ambient temperature for 20 min. Thereafter, each tissue section was subjected to 24 h of primary and biotinylated secondary antibody incubation under 4°C (1:1000, Beyotime Biotechnology). Later, positively stained cells were measured using hematoxylin and DAB reagent. Primary antibodies used are as follows: Anti-ADAM9 antibody (Abcam, ab186833, 1:50), Anti-SQSTM1/p62 antibody (Abcam, ab109012,1:50), Anti-Bax (Abcam, ab32503, 1:250). Finally, the stained cells were observed at ×200 or ×400 using Leica QWin Plus v3 software under the same parameter setting [16].

### 2.12. Statistical analysis

SPSS22.0 was utilized to statistically analyze experimental data. We displayed the measurement data in the form of mean ± SD (x ± s). Results were compared using one-way ANOVA. p < 0.05 stood for statistical significance.

## 3. Results

In this work, we found that ADAM9 decreased HCC radiosensitivity, autophagy mediated this process. We also identified a crucial role of Nrf2 in ADAM9 promoting autophagy. The level of ADAM9 expression in HCC cells increased after X-ray irradiation. Through CCK-8 assays, subcutaneous tumorigenesis experiments, and clone formation assays, this work discovered the increased radiosensitivity of MHCC97H cells after ADAM9 knockdown, and the radiosensitivity of Huh7 cells decreased after the overexpression of ADAM9. Furthermore, ADAM9 induced HCC cell autophagy through downregulating Nrf2 expression, while autophagy inhibition or induction reversed the effects of ADAM9 on radiosensitivity. ADAM9 level showed a negative correlation with Bax and p62 expression within HCC tissues after radiotherapy.

### 3.1. Radiation increases the level of ADAM9 in HCC cells

**Figure 1.**
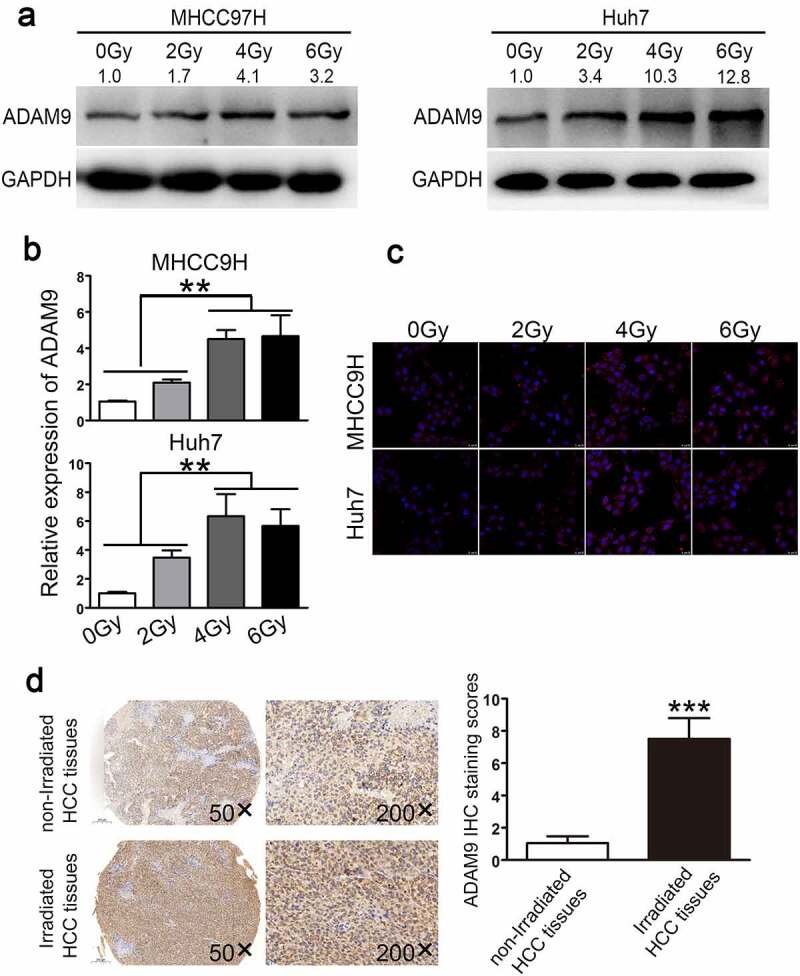
ADAM9 expression within HCC cells and tissues under diverse radiotherapy conditions. (a),(b)qRT-PCR and western blot assays were conducted for detecting ADAM9 protein and mRNA expression in MHCC97H cells and Huh7 cells after irradiated with X-ray at diverse doses. (c) ADAM9 up-regulation was verified through immunofluorescence staining of HCC cells following irradiation at 2, 4, 6 Gy as well as 24 h of incubation. (d) Typical image for ADAM9 immunohistochemical staining of HCC samples treated with or without radiotherapy. 5 randomized regions were observed under light microscopy (200× total magnification). The staining score data are shown as means ± SDs. * * *P < 0.001

ADAM9 expression in Huh7 and MHCC97H cells that showed poor or high malignancy grade was investigated following irradiation. According to Figure 1(a), ADAM9 protein expression was affected by irradiation at doses of 0, 2, 4, and 6 Gy depending on the dose, and ADAM9 levels were notably up-regulated as irradiation dose increased. ADAM9 levels increased to approximately 1.7-, 4.1-, and 3.2-fold, respectively, at doses of 2, 4, and 6 Gy compared to those of the non-treated corresponding controls in MHCC97H cells, while induced to about 3.4-, 10.3-,12.8-fold in Huh7 cells. These data were confirmed by qRT-PCR (Figure 1(b)). The confocal microscopy results suggested increased ADAM9 level within cell cytoplasm after exposure to irradiation (Figure 1(c)). Two portions of HCC specimens were adopted to define the association of radiation with ADAM9 level. As observed from Figure 1(d), ADAM9 level of the radiation group significantly increased relative to non-radiation group, which suggested that ADAM9 level was positively related to radiation.

### 3.2. ADAM9 influenced HCC cell radiosensitivity

**Figure 2.**
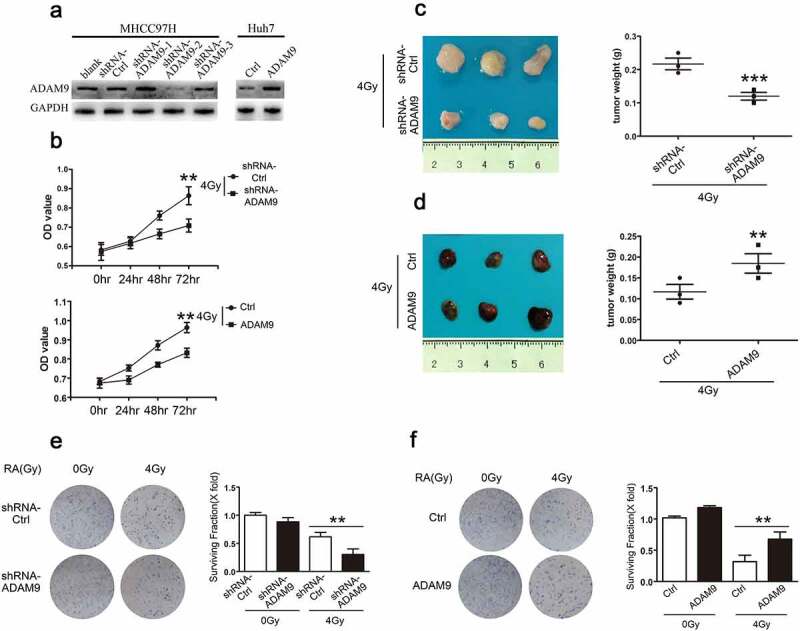
Role of ADAM9 in HCC cell radiosensitivity. (a) Western blot analysis showed the ADAM9 silencing within MHCC97H cells as well as ADAM9 over-expression within Huh7 cells. (b) The cell survival rate was determined by the CCK-8 method after 0, 24, 48 and 72 h of 4 Gy X-ray irradiation exposure at 4 Gy. (c), (d) Proliferation of MHCC97H cells and Huh7 cells following X-ray radiation at 4 Gy was detected by subcutaneous tumor formation in nude mice. (e), (f) Colony formation experiments were conducted for detecting MHCC97H cell and Huh7 cell radiosensitivity after changing ADAM9 expression. Data were displayed in a form of mean ± SD from 3 independent assays conducted in triplicate.*p < 0.05,**p < 0.01, and***p < 0.001

shRNA successfully induced ADAM9 silencing of MHCC97H cells (MHCC97H-shRNA-ADAM9) and the stable ADAM9 over-expression within Huh7 cells (Huh7-ADAM9), as verified through western blot experiments (Figure 2(a)). We also conducted CCK-8 assays for detecting the role of ADAM9 level in cell viability at 0, 24, 48 and 72 h following X-ray irradiation at 4 Gy. According to Figure 2(b), relative to control, the MHCC97H-shRNA-ADAM9 cell group had a lower OD value, while the Huh7-ADAM9 cell group had a higher OD value, suggesting that ADAM9 could increase cell viability after irradiation. Furthermore, for assessing the ADAM9 role in HCC cell tumorigenicity after irradiation, subcutaneous tumor formation experiments in nude mice were conducted. As shown in Figure 2(c), after 4 Gy X-ray irradiation, the MHCC97H-shRNA-Ctrl and MHCC97H-shRNA-ADAM9 groups were subcutaneously inoculated into male nude mice. Compared with the MHCC97H-shRNA-Ctrl cell group, the MHCC97H-shRNA-ADAM9 cell group exhibited significantly reduced tumor volume and weight. Accordingly, the Huh7-ADAM9-generated xenograft size remarkably increased relatively to Huh7-ctrl-obtained xenografts after 4 Gy X-ray irradiation (Figure 2(d)). These data indicated that ADAM9 could significantly attenuate the radiosensitivity of HCC cells. Moreover, colony formation assay was performed to confirm this conclusion. We found that the cells with higher ADAM9 expression showed a decreased survival rate after 4 Gy X-ray irradiation. Taken together, these data suggested that ADAM9 reduced HCC cell radiosensitivity both in vivo and in vitro.

### 3.3. ADAM9 decreases the radiosensitivity of HCC cell lines by inducing autophagy

**Figure 3.**
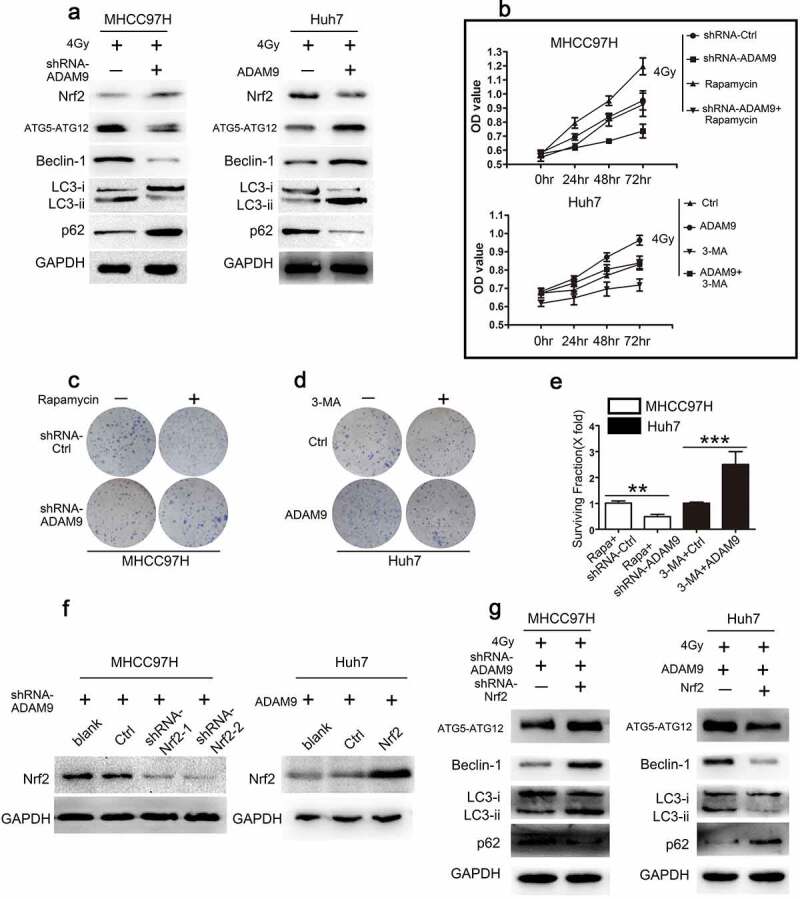
ADAM9 decreased radiosensitivity of HCC cells via inducing autophagy. (a) Autophagy-related protein levels in MHCC97H and Huh7 cells were detected by western blot assays after changing ADAM9 expression. (b) The cell viability was determined by the CCK-8 method after 0, 24, 48 and 72 h of 4 Gy X-ray irradiation exposure in different groups. (c, d, e) Clone formation experiments were conducted in different groups after 4 Gy X-ray irradiation. (f) Western blot showed screening of MHCC97H cells with Nrf2 downregulation and Huh7 cells with Nrf2 upregulation. (g) Autophagy related protein expression after knockdown of Nrf2 in ADAM9-downregulated MHCC97H cells or upregulation of Nrf2 in ADAM9-overexpressing Huh7 cells. Data were displayed in a form of mean ± SD from 3 independent assays conducted in triplicate.*p < 0.05,**p < 0.01, and***p < 0.001

In order to evaluate autophagic activity, the autophagy-specific markers, including p62, LC3 II, LC3 II/LC3 I ratio, Beclin-1, ATG5-ATG12 were detected by western blot analysis. According to Figure 3(a), ADAM9 knockdown within MHCC97H cells reduced LC3 II/LC3 I ratio and LC3 II, Beclin-1, AT5-ATG12 expression, and increased p62 protein expression. Conversely, following the transfection of ADAM9 into Huh7 cells, the LC3 II, Beclin-1, AT5-ATG12 protein levels and the LC3 II/LC3 I ratio elevated, whereas p62 protein levels declined. Based on these findings, ADAM9 promoted autophagy in HCC cells. We speculated that autophagy may be involved in the process that ADAM9 reduces HCC cell radiosensitivity. To elucidate the autophagy effects on ADAM9-mediated cell radiosensitivity regulation, rapamycin, a well-known activator of mammalian autophagy, was used to treat MHCC97H cells, and 3-MA, a well-known inhibitor of autophagy, was adopted for treating Huh7 cells. According to Figure 3(b), MHCC97H cells were transfected using ADAM9-shRNA, treated with rapamycin or both. After 4 Gy X-ray irradiation, CCK-8 assays were used to detect the cell viability of different groups. As revealed by these findings, cell viability decreased after ADAM9-shRNA transfection relative to controls. Treating cells with ADAM9-shRNA and rapamycin alleviated the decrease induced by ADAM9-shRNA transfection. The viability of the MHCC97H cells treated with rapamycin alone was increased. Huh7 cells were transfected with ADAM9, treated with 3-MA or both. After 4 Gy X-ray irradiation, CCK-8 assays were used to detect the cell viability of different groups. As a result, cell viability elevated after ADAM9 transfection relative to controls. Treating cells with ADAM9 and 3-MA alleviated the increase induced by ADAM9 transfection. The viability of the Huh7 cells treated with 3-MA alone was decreased. Furthermore, in Figure 3(c-e), we found that the decreased survival rate of MHCC97H cells induced by ADAM9 deficiency was inhibited by rapamycin. 3-MA also alleviated the upregulation of the survival rate induced by ADAM9 in Huh7 cells. These findings demonstrated that ADAM9 decreased radiosensitivity in HCC cells by inducing autophagy. To further determine the underlying mechanism in the process of ADAM9 inducing autophagy in HCC cells, the role of nuclear factor erythroid 2-like 2 (Nrf2) was explored. As in Figure 3(a), Nrf2 expression in the cytoplasm increased after knockdown of ADAM9 in MHCC97H cells. Conversely, Nrf2 level elevated in Huh7-ADAM9 cells. Subsequently, we screened Nrf2-downregulated MHCC97H cells and Nrf2-upregulated Huh7 cells by Western blot as shown in Figure 3(f). The knockdown of Nrf2 induced LC3 II, LC3 II/LC3 I ratio, Beclin-1, AT5-ATG12 and reduced p62 in MHCC97H-shRNA-ADAM9 cells after 4 Gy X-ray irradiation. Consistent with these changes, the overexpression of Nrf2 in Huh7-ADAM9 cells, reduced LC3 II, LC3 II/LC3 I ratio, Beclin-1, AT5-ATG12 and induced p62 expression (Figure 3(g)). Combined with Figure 3(a), we found that changing of autophagy markers induced by ADAM9 could be alleviated by Nrf2 expression. These results suggested that ADAM9-induced autophagy were mediated by Nrf2.

### 3.4. Correlation between ADAM9 expression and BAX, p62 levels within HCC samples after radiotherapy

**Figure 4.**
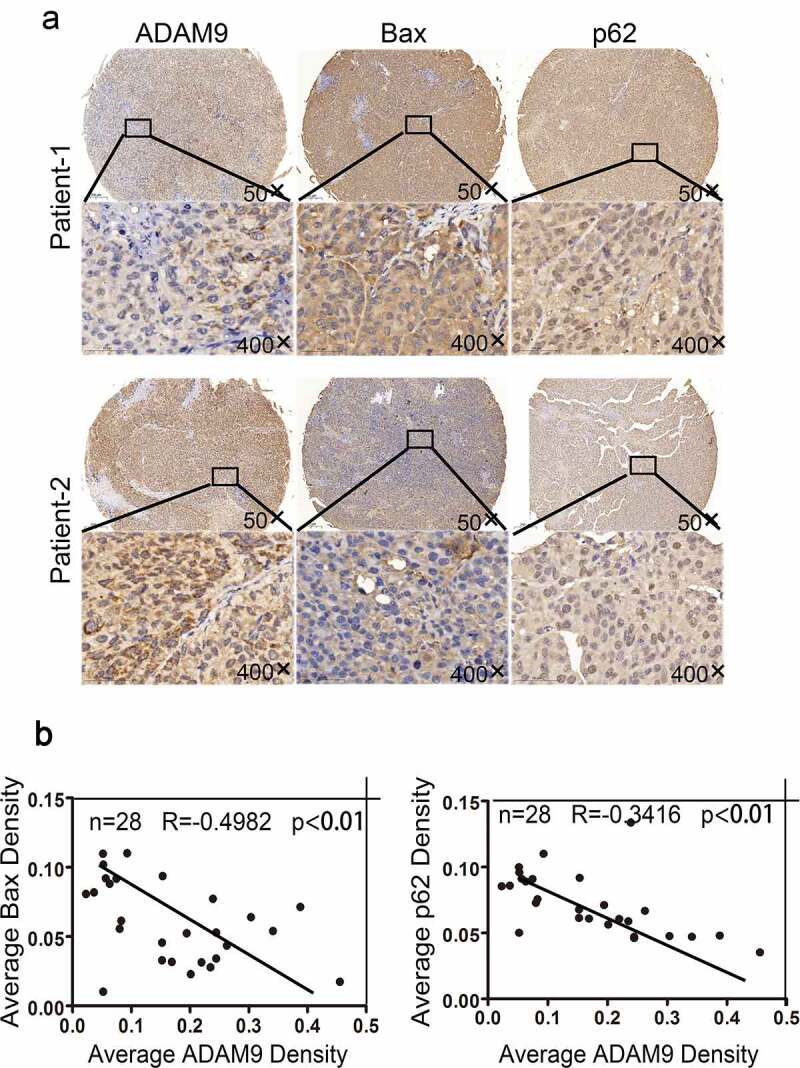
Correlation between ADAM9 expression and BAX and p62 expression in HCC tissues after radiotherapy. (a) Immunohistochemical staining was conducted for determining the association of ADAM9 expression with autophagy-related P62 and Bax protein expression within human HCC samples. (b) Results of multiple linear regression analysis of ADAM9 expression and BAX and P62 expression. Data were displayed in a form of mean ± SD from 3 independent assays conducted in triplicate.*p < 0.05,**p < 0.01, and***p < 0.001

To define the association of ADAM9 expression with cell apoptosis and autophagy, 28 HCC tumor tissue samples were collected after radiotherapy. Figure 4(a) displays the IHC staining examples. According to the results, ADAM9 up-regulation decreased Bax and p62 (Figure 4(a)). As revealed by multiple-linear regression analysis, ADAM9 level showed negative correlation with p62 (R = −0.3416, p < 0.01) and Bax (R = −0.4982, p < 0.01) expression (Figure 4(b)). Together, the above results showed that ADAM9 may inhibit apoptosis and induce autophagy in HCC tissues after radiotherapy.

## 4. Discussion

ADAM9, a metallozinc proteinase expressed on the cell surface, predicted dismal prognostic parameters as well as short survival times in patients with various cancers [23,24]. Several studies have found that ADAM9 is overexpressed in solid tumors, including gastric cancer (GC), prostate cancer (PCa), liver cancer, melanoma, pancreatic ductal adenocarcinoma, breast cancer (BC), glioma, and lung cancer, providing a new target for the treatment of tumors [23–29]. Radiotherapy accounts for a major approach for treating malignant tumors, and the sensitivity of tumor cells to radiotherapy is tightly associated with patient survival [30]. Although the ADAM9 effect on human tumors has been extensively studied, the relationship between ADAM9 and HCC cell radiosensitivity, as well as the underlying mechanism, has not been established. Therefore, this is the first study to explore the potential effect of ADAM9 on HCC cell radiosensitivity and its possible mechanism.

The expression of ADAM9 in cancer cells can be modulated via certain factors. Study showed that circ_0020123 can inhibit ADAM9 expression through the release of the sponge miR-488-3p in NSCLC cells [31]. ADAM9 was also confirmed to be a target gene binding to circ_0005276, and its level was positively regulated by circ_0005276 and could exacerbate the development of epithelial ovarian cancer [32]. Numerous studies have shown that ADAM9 is negatively regulated via miRNAs within HCC, and miR-126 [33], miR-203 [34], and miR-488 [35] can target ADAM9 3'-UTR and downregulate its expression for suppressing cell invasion and migration in vitro. Sorafenib and regorafenib are multireceptor tyrosine kinase inhibitors and clinical drugs used in treating HCC. ADAM9 level is inhibited by these drugs, leading to the upregulation of mMICA, thereby enhancing the activity of natural killer cells against HCC [13,36]. However, the effect of irradiation on ADAM9 expression has not been reported thus far. In this study, qRT-PCR, IF and WB assays were utilized for exploring ADAM9 level in HCC cells after X-ray irradiation. As a result, ADAM9 mRNA and protein expression increased. Then, we hypothesized that the HCC cell radiosensitivity was related to ADAM9 level to some extent.

Despite advances in radiotherapy, radiation resistance remains a challenge. To improve sensitivity to radiation, researchers have focused on improving hypoxia, increasing DNA damage, and influencing the cell cycle [37]. For example, previous studies have shown that changing hypoxia can alter radiosensitivity, and hypermethylation possibly influences cancer radiosensitivity through down-regulating reoxygenation-associated genes [37]. Clinical radiobiological effects are determined by five factors, namely the 5 Rs of radiobiology, which include DNA repair, reproliferation, reoxygenation, redistribution, and radiosensitivity, which determine the efficacy of tumors [38]. The radiosensitivity of MHCC97H cells and Huh7 cells under 4 Gy irradiation was measured through colony formation, subcutaneous tumor formation and CCK-8 assays within nude mice. As a result, ADAM9 promoted HCC cell viability and tumorigenicity after X-ray irradiation, suggesting that ADAM9 could reduce the radiosensitivity of HCC cells. Consistent with these results, Josson found that ADAM9 silencing led to enhanced radiosensitivity and apoptosis of human prostate cancer cells [39].

The exact molecular mechanisms related to the cell-signaling network that links ADAM9 with radiosensitivity have been greatly unclear. Our study suggested that autophagy mediated the mechanism by which ADAM9 decreased the radiosensitivity of HCC cells. Due to the different intensities of autophagy, the regulatory effect of autophagy on cell radiosensitivity is also different. Some scholars believe that when the autophagic ability of cells is too strong, organelle or key protein degradation by autophagy exceeds cell compensation, leading to cell death. Thus, autophagy potentially promotes cancer cell radiosensitivity. Therefore, many studies have used drugs or gene interventions to enhance the autophagy induced by X-ray irradiation to promote tumor cell death and enhance tumor cell radiosensitivity. In glioma stem cells, nvp-bez235 elevated glioma stem cell radiosensitivity in vitro through the activation of autophagy [40]. Other researchers believe that autophagy can exert an important part in cell self-protection and maintain cell metabolism balance. In this case, autophagy reduces cancer cell radiosensitivity and enhances their resistance to radiotherapy. Mitrakas AG et al. silenced the LC3A, LC3B and TFEB genes and found that glioblastic tumor cells and xenografts in mice were more sensitive to radiation, which indicated that autophagy could reduce the sensitivity of cell emission [41]. As a commonly used inhibitor of autophagy, 3-MA can inhibit the occurrence of autophagy. Related studies have used 3-MA to interfere with esophageal cancer cells (EC9706), confirmed that radiation-induced autophagy protects against cell death, and inhibiting autophagy can promote esophageal squamous cell carcinoma (ESCC) radiosensitivity [42]. Some scholars found that after using 2-propylpentanoic acid (VPA) to inhibit Histone deacetylase (HDAC) function, autophagy was inhibited, and cell radiosensitivity was enhanced [43].

Underlying mechanism in ADAM9 inducing autophagy was explored. In several studies, PI3K/Akt/mTOR signaling pathway is involved in regulating autophagy [44]. In the previous study, ADAM9 could enhance ROS generation. Nuclear factor erythroid 2-like 2 is an important transcriptional regulatory factor, which is activated by ROS to drive the expression of antioxidant genes and protect from oxidant injury [45]. The Nrf2 regulatory gene includes a group of antioxidant enzymes. Under quiescent conditions, Nrf2 binds to cytoplasmic Keap1, leading to proteasomal degradation. Cell stimulations, such as oxidative stress, induces conformational changes in Keap1, lead to the release of Nrf2. Nrf2 was then transposed to the nucleus and inactivated gene expression containing antioxidant elements in its promoter region. In the study, ADAM9 reduced Nrf2 expression in the cytoplasm of HCC cells. It has been reported that transcription factor Nrf2 regulates the autophagic signaling pathway by modulating adaptor protein p62/SQSTM, which serves as a selective autophagy substrate [46]. In the research, ADAM9 inducing autophagy mediated by Nrf2.

Many articles have suggested that cell radiosensitivity is associated with apoptosis [47,48]. Radiotherapy can slow the growth of tumor cells by inducing apoptosis. There are two kinds of apoptosis-regulating genes, namely, proapoptotic and antiapoptotic genes; of these genes, Bax and Bcl-2 are the most representative proapoptotic protein and antiapoptotic protein, respectively. As revealed by previous experiments, Bcl-2-to-Bax ratio affects apoptosis. Mackey concluded in some experiments that the higher Bcl-2-to-Bax ratio results in the higher the chance of radiotherapy failure in prostate cancer patients [49]. As discovered by Sirui Li and colleagues, lung cancer cell radiosensitivity was enhanced by upregulating Bax and downregulating Bcl-2 [50]. In triple-negative breast cancer (TNBC), TMPRSS4 inhibited TNBC apoptosis induced by 6 Gy IR, and the expression of Bax and Caspase 3 was overexpressed, while the expression of Bcl2 was downregulated, and radiotherapy sensitivity was decreased [51]. Our study found that apoptosis may be another mechanism by which ADAM9 inhibits cell radiosensitivity. Studies by Xiaoyun Wang et al. showed that recombinant ADAM9 increased apoptosis by reducing the activation of Akt survival pathway [52].

Taken together, our study first illustrates the influence of ADAM9 on liver cancer cell radiosensitivity. The data strongly suggest that ADAM9 decreases HCC cell radiosensitivity through the induction of autophagy. ADAM9 over-expression within HCC accounts for the potent indicator of insensitivity to X-ray irradiation. The relevant mechanisms need to be further explored. Such studies will increase our understanding of the radiosensitivity of HCC cells and help us identify new therapeutic targets.

## 5. Conclusion

X-ray irradiation promoted ADAM9 expression, which could decrease the HCC radiosensitivity. ADAM9 decreased HCC radiosensitivity by regulating autophagy, which provides a new target for enhancing the curative effect of liver cancer radiotherapy.

## Data Availability

Datasets utilized during this work can be accessed from related authors after reasonable request.

## Highlights

- Radiation increases the level of ADAM9 in HCC cells.
- ADAM9 decreases the radiosensitivity of HCC cell lines by inducing autophagy.
- ADAM9 induced autophagy via downregulating Nrf2 expression in the cytoplasm.
